# The etiology of uracil residues in the *Saccharomyces cerevisiae* genomic DNA

**DOI:** 10.1007/s00294-018-0895-8

**Published:** 2018-10-17

**Authors:** Norah Owiti, Kasey Stokdyk, Nayun Kim

**Affiliations:** 1 Department of Microbiology and Molecular Genetics, University of Texas Health Science Center at Houston, Houston, TX 77030 USA; 2 MD Anderson Cancer Center UT Health Graduate School of Biomedical Sciences, Houston, TX 77030 USA; 3 Present Address: Department of Biological Engineering, Massachusetts Institute of Technology, Cambridge, MA 02139 USA

**Keywords:** DNA repair, Non-canonical nucleotides, Transcription-associated mutagenesis, Uracil, dUTPase

## Abstract

Non-canonical residue in DNA is a major and conserved source of genome instability. The appearance of uracil residues in DNA accompanies a significant mutagenic consequence and is regulated at multiple levels, from the concentration of available dUTP in the nucleotide pool to the excision repair for removal from DNA. Recently, an interesting phenomenon of transcription-associated elevation in uracil-derived mutations was described in *Saccharomyces cerevisiae* genome. While trying to understand the variability in mutagenesis, we uncovered that the frequency of uracil incorporation into DNA can vary depending on the transcription rate and that the non-replicative, repair-associated DNA synthesis underlies the higher uracil density of the actively transcribed genomic loci. This novel mechanism brings together the chemical vulnerability of DNA under transcription and the uracil-associated mutagenesis, and has the potential to apply to other non-canonical residues of mutagenic importance.

## Introduction

Uracil, a very frequent form of endogenous DNA modification, can appear in DNA via two different mechanisms: the incorporation into DNA in place of thymine and the deamination of cytosine. Many DNA polymerases including eukaryotic replicative DNA polymerases cannot distinguish between a uracil and a thymine base, and will readily incorporate uracil in place of thymine during replication and repair, depending on the [dUTP]/[dTTP] ratio, resulting in a stable U:A base pair (Bessman et al. 1958; Warner et al. 1981). If uracil persists to the subsequent round of replication, adenine can be incorporated in the complementary strand; U incorporation in place of T, therefore, is not mutagenic in itself. The cytosine deamination can occur either through a spontaneous or an enzymatic process creating U:G mispairs, which potentially is highly mutagenic. If uracil persists in a U:G mispair, the subsequent round of replication would result in a C:G-to-T:A conversion. On the other hand, if an abasic site is generated through the removal of uracil by a uracil DNA glycosylase, it can either be repaired in error-free manner by base excision repair machinery or result in further mutagenic event mediated by the error-prone translesion synthesis DNA polymerases (Boiteux and Jinks-Robertson 2013). Repeated cycles of uracil excision by glycosylases also come with the risk of the accumulation of abasic sites leading to DNA strand breaks and, ultimately, cell death (el-Hajj et al. 1988; Gadsden et al. 1993). It is, thus, important to maintain the minimal level of uracil in DNA to prevent genomic instability. Here, we discuss the wider implication of a novel mechanism of uracil occurrence in DNA that is recently reported by our lab.

### The sources of uracil in DNA

Even though uracil in DNA has been indicated to be a major source of genomic instability, we still lack information on the number of uracil DNA residues derived from either deamination and/or incorporation. Various quantification methods aimed at measuring the genome-wide level of uracil in DNA have yielded inconsistent results and have the added complication that the quantification methods cannot distinguish U:A and U:G base pairs, arising from uracil incorporation and cytosine deaminations, respectively (Galashevskaya et al. 2013; Horvath and Vertessy 2010). Spontaneous deamination of cytosines is estimated to occur about 70–500 times per cell per day (Kavli et al. 2007; Lindahl 1993). In addition, enzymes that deaminate cytosines to uracil, such as Alipoprotein B mRNA-editing enzyme catalytic polypeptide-like family proteins (APOBECs) including Activation Induced Deaminase (AID), have been identified in many metazoan species (Siriwardena et al. 2016). These enzymes are critical to antibody-diversification and innate immunity against retroviruses. It is, therefore, possible that the number of uracil residues that are derived from cytosine deamination in metazoa is significantly higher than others without clearly characterized cytosine deaminases.

**Fig. 1:**
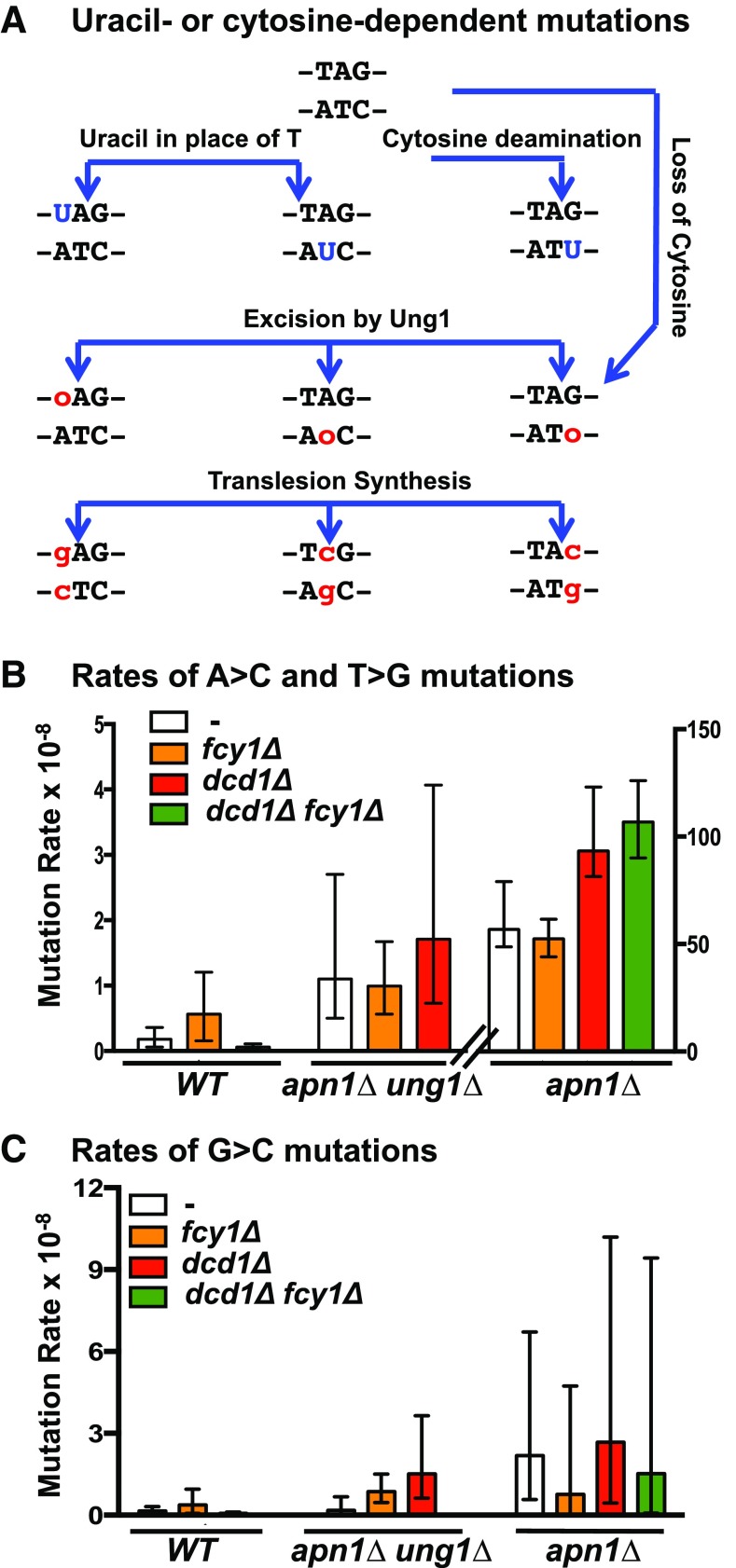
Uracil- or cytosine-dependent mutations at the *pTET-lys2-TAG* reporter. **a** Excision of uracil from U:A base pairs (U incorporated in place of thymine) or from U:G base pairs (U generated by deamination of C) result in T>G/A>C or G>C mutations, respectively, by the insertion of C nucleotide opposite AP sites by the TLS polymerases. **b, c** Rates of mutation for each indicated strain were calculated from 12 to 24 independent cultures using the method of median. For each strain, 48–90 independent mutants were sequenced to determine the types of mutations occurring at the TAG stop codon. Error bars represent the 95% confidence intervals. Non-overlapping error bars are considered statistically significant and vice versa. **b** Rates of mutations resulting from the excision of uracil incorporated in place of thymine on the transcribed and non-transcribed strand (A>C and T>G, respectively). The Y-axis on the left applies to *WT* and *apn1Δ ung1Δ* values and the *Y*-axis on the right is for *apn1Δ* values. **c** Rates of mutations resulting from the loss of cytosine base on the transcribed strand (G>C)

On the other hand, in yeast, a large fraction of genomic uracil is thought to originate from the dUTP incorporation by DNA polymerases rather than from cytosine deamination. Accordingly, the excision of uracil base-paired to an adenine rather than to a guanine is a major source of abasic sites in the yeast genome (Guillet and Boiteux 2003). Our lab has contributed toward estimating the number of uracils in DNA by studying the mutations originating from cytosine deamination and uracil incorporation in the *S. cerevisiae* model system using several well-defined mutation reporters, which can be expediently screened for mutation via a change in nutrient requirement (Kim and Jinks-Robertson 2009; Kim et al. 2011b; Owiti et al. 2018). One of these reporters, the *pTET-lys2-TAG*, reverts by changes to the TAG stop codon inserted in-frame into the *LYS2* gene, which encodes an enzyme essential in lysine biosynthesis. The AP sites, which are generated by the excision of uracil base by Ung1 DNA glycosylase, are bypassed with the insertion of mostly Cs in the complementary strand by the TLS polymerases. At a TAG stop codon, the overall mutational output resulting from uracil, incorporated in place of thymine on either DNA strand or resulted from the cytosine deamination, are T>G/A>C or G>C, respectively (Fig. 1a). The abasic sites generated by the spontaneous loss of cytosine base can also be a contributing factor for G>C mutations. In *apn1∆* background, where the error-free repair of AP lesions is largely disabled, the rate of uracil-dependent (T>G/A>C) mutations is ~ 20-fold higher than the cytosine-dependent (G>C) mutations (Fig. 1b, c). This dramatic difference between the uracil and the cytosine-associated mutations was consistently observed when the deoxycytidine monophosphate deaminase, Dcd1, or the putative cytosine deaminase, Fcy1, was disrupted in *apn1∆ dcd1∆* or *apn1∆ fcy1∆* strain, respectively. In fact, the disruption of Fcy1, which was recently reported to facilitate deamination of cytosines that are located in the chromosomal DNA (Freudenreich 2018, Su and Freudenreich 2017), did not significantly change the rate of G>C mutations in *apn1*∆ or *apn1∆ dcd1∆* background. The rates of G>C mutations were also not significantly affected by the disruption of the uracil DNA glycosylase, Ung1, whereas the rates of T>G/A>C mutations were greatly reduced in all backgrounds. Together, these data suggest that, in the context of our reporter assay, a majority of genomic uracil in yeast originate from the incorporation of uracil into DNA by DNA polymerases rather than from cytosine deamination. This finding is rather restricted by the limitation of the *pTET-lys2-TAG* system and needs further experiments to see if it generally applies. For example, in the reporter system used here, the only surveyed cytosine base is located on the transcribed DNA strand annealed to the nascent mRNA and, therefore, not single-stranded during transcription. The mammalian cytosine deaminase AID has a strong preference for the cytosines located within the context of single-stranded DNA (Bransteitter et al. 2003; Chaudhuri et al. 2003), and this might be also true for the yeast cytosine deaminases.

### Free dUTP pools and incorporation of uracil into DNA

In general, circumstances that would increase [dUTP]/[dTTP] ratio would increase the incorporation of uracil into DNA. Studies have shown that exogenously treating yeast cells with 5-fluorouracil (5-FU), a thymidylate synthase (TS) inhibitor, increase the amount of uracil incorporated into DNA (Owiti et al. 2018; Seiple et al. 2006). By inhibiting TS, 5-FU blocks dTTP synthesis and, therefore, increases the [dUTP]/[dTTP] ratio. Modulating the levels of enzymes involved in the dTTP biosynthesis process can also affect the [dUTP]/[dTTP] ratio and facilitate uracil incorporation. Although the dUTP pyrophosphatase Dut1 is the major enzyme responsible for the synthesis of dUMP, the obligate precursor of dTTP synthesis, dUMP can also be synthesized by another highly conserved enzyme deoxycytidine monophosphate deaminase, Dcd1, which converts dCMP to dUMP (Wang and Weiss 1992). The dUMP production by Dcd1, albeit much less robust than that by Dut1, is sufficient in generating adequate dTTP to sustain replication (McIntosh et al. 1986). Studies in *S. cerevisiae* indicated that deletion of *DCD1* led to a significant increase in dCTP and reduction in dTTP pool without affecting the viability of the cells (Kohalmi et al. 1991; Sanchez et al. 2012). An increase in mutagenesis derived from uracil following the deletion of *DCD1* gene was previously reportedand confirmed by our own investigation using the *pTET-Lys2-TAG* reporter (Fig. 1b, c) (Kohalmi et al. 1991; Owiti et al. 2018). The level of uracil-associated mutations in yeast cells is, therefore, highly sensitive to the fluctuation in the [dUTP]/[dTTP] ratio and likely correlates with the frequency of dUTP usage by DNA polymerases.

### Non-uniform distribution of uracil in DNA

Recent studies suggest that the uracil distribution in the genome is unexpectedly non-uniform with several factors dictating what parts of the genome serve as hotspots of uracil. (Bryan and Hesselberth 2015; Kim and Jinks-Robertson 2009; Owiti et al. 2018; Shu et al. 2018). Earlier work using yeast genetic approach showed an increase in the rate of mutations with a distinct uracil-associated signature (A:T-to-C:G transversions). These mutations were dramatically reduced when the uracil DNA glycosylase, Ung1, was disabled or when the yeast dUTPase, Dut1, was overexpressed further supporting the hypothesis that the rate of A:T-to-C:G mutations can be correlated with the frequency of uracil in DNA. More interestingly, these mutations were almost eliminated when the transcription of the reporter gene was suppressed, indicating that transcription can dictate the extent of uracil content in the genomic DNA in a locus-specific manner.

Our recent work extended the investigation by directly quantifying uracil in DNA at several different loci in the yeast genome to confirm the previous mutagenesis experiments (Owiti et al. 2018). Because the measurement of uracil in DNA is complicated by the similarity of uracil and thymine, we adapted the long-amplicon qPCR approach that was successfully used to quantify DNA damage at different parts of the genome (Ayala-Torres et al. 2000; Horvath and Vertessy 2010; Hunter et al. 2010). Using the long-amplicon qPCR technique, we revealed that, at a single gene with regulatable promoter, the uracil density varied by > twofold depending on the transcription rate (Table 1). Comparing the highly transcribed *TDH3* gene to the moderately transcribed *CAN1* gene also reiterated the correlation between transcription rate and the uracil density in the yeast genome. This observation of non-random distribution of uracil content has also been implied by other reports. Replication timing was indicated to be a determinant of uracil DNA content in yeast and *E. coli* (Bryan et al. 2014). This study showed that the early and late replication origins are completely depleted of uracil residues and that altering nucleotide biosynthesis disrupts the regulation of uracil incorporation into DNA. Another genome-wide study of uracil content in the human genome found that uracil is not randomly distributed throughout the genome and enriched in the centromere regions (Shu et al. 2018). Together with our recently published result, these studies show that a non-uniform distribution of uracil across the genome is a conserved feature from prokaryotes to metazoans. The significance of this disproportionate pattern of uracil density and specifically the correlation with transcription rate at the genome-wide scale warrant further investigation.

**Table 1:** Density of uracil in DNA correlates with the transcription rate

	*pTET-lys2* (transcription OFF)	*pTET-lys2* (transcription ON)
Transcription level*	0.51 ± 0.14	87.9 ± 14.8
Uracil-associated mutations**	0.29	38
Uracil residues/10Kb***	0.64 ± 0.29	1.31 ± 0.2

	*CAN1* (transcription LOW)	*TDH3* (transcription HIGH)
Transcription level*	9.6 ± 1.5	1066 ± 461
Uracil residues/10Kb***	0.26 ± 0.155	0.98 ± 0.35

The data presented here were previously reported in Kim and Jinks-Robertson (2009) and Owiti et al. (2018)

*Transcription levels were determined by qRT-PCR and are normalized to the level of transcription of *ALG9*

**The rates of uracil-associated mutations (× 10^− 9^) were calculated in *apn1∆* cells with (OFF) or without (ON) doxycycline

***The numbers of uracil residues per 10 Kb were derived from the amplification efficiency measured using the long-amplicon PCR approach

**Fig. 2:**
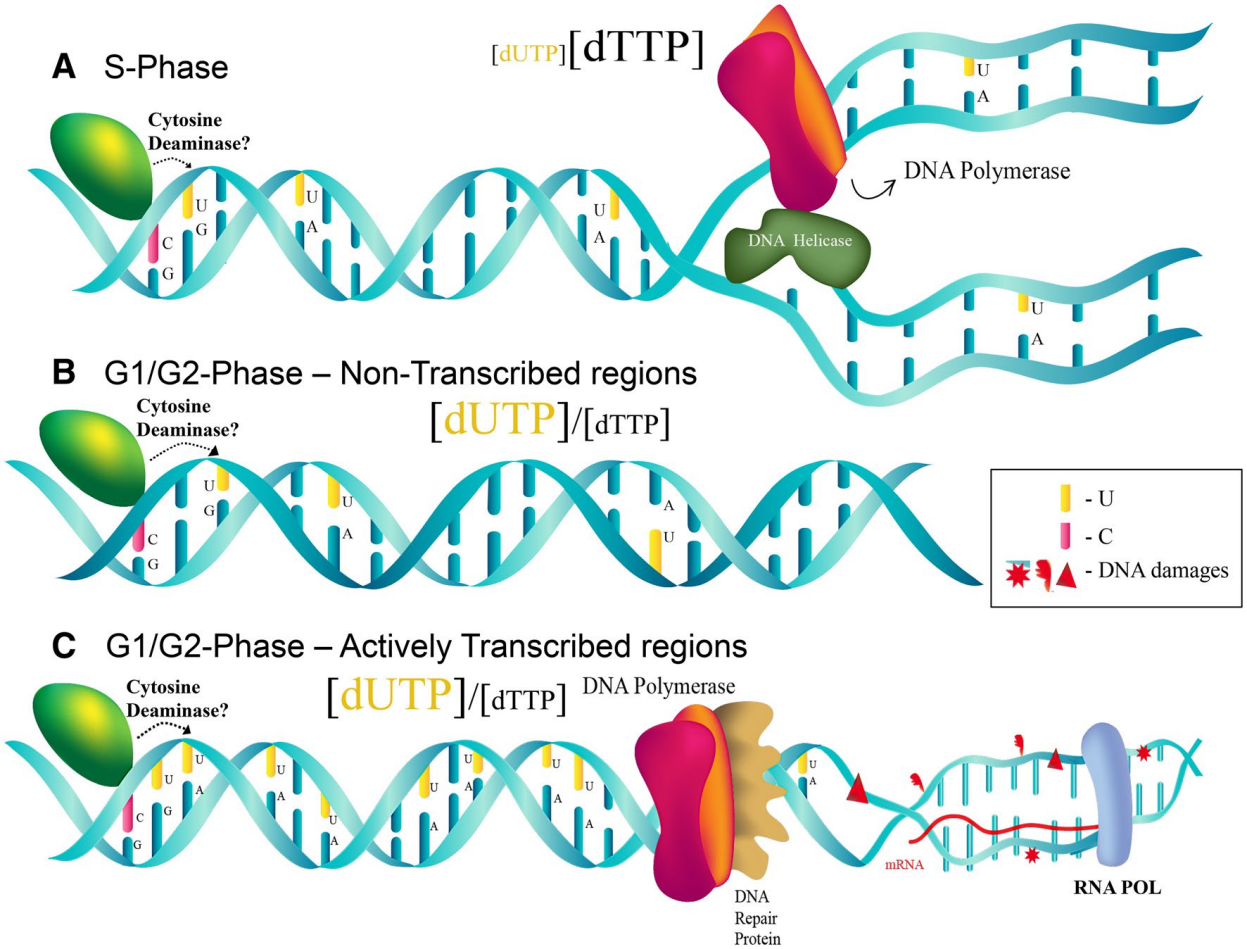
Model of repair-associated incorporation of uracil into DNA

We have also gained some insight into the mechanism of transcription-associated elevation in uracil density. The dUTP pool in G1 and G2 is significantly higher compared to S phase, because the expression of dUTPase-encoding gene is significantly induced in the S-phase, ensuring minimal [dUTP] during replication (Cho et al. 1998; Ladner and Caradonna 1997; Pardo and Gutierrez 1990). For the DNA synthesis occurring outside S-phase, such as that associated with repair, the available nucleotide pool has a relatively higher [dUTP]/[dTTP], which translates into the higher risk of incorporating uracil into DNA during the repair synthesis occurring during G1 or G2. Interestingly, reducing the [dUTP]/[dTTP] ratio by overexpressing dUTPase from G1- or G2-specific promoters significantly reduced the rate of uracil-associated mutations only at the highly transcribed mutation reporter. At highly transcribed genomic loci, that is, more frequent rounds of DNA repair synthesis occurring in G1 and G2 could be the key events leading to the higher uracil DNA content (see model in Fig. 2). This model of uracil incorporation during repair synthesis is further supported by the elevation in uracil-dependent mutations and uracil residues in the genome observed when cells are treated with DNA damaging agent CPT or 4NQO, without any reported connection to the dUTP/dTTP metabolic pathway. Underlying this model is the assumption that active transcription will lead to higher level of DNA damage necessitating DNA repair synthesis in G1 and G2. Although we were not able to directly demonstrate such repair synthesis occurs, there are many previous reports, indicating that DNA under active transcription is generally more susceptible to base damages and other chemical modifications (reviewed in (Jinks-Robertson and Bhagwat 2014; Kim and Jinks-Robertson 2012)). Active transcription is also reported to facilitate the formation of non-canonical secondary structures such as G4-DNA or R-loops, which can be recognized as DNA damages and elicit DNA damage response (Fan et al. 2018). It would be important to determine whether DNA synthesis associated with resolving such structures could also contribute to the increased incorporation of uracil into DNA. In addition, there are reports of a close connection between DNA damage response/DNA repair pathways and co-transcriptional, nascent RNA processing mechanisms (Mikolaskova et al. 2018). Nonetheless, the analysis of unscheduled DNA synthesis occurring at actively transcribed regions of the genome in cells not treated with exogenous genotoxic agents is an important and necessary next step.

### Non-uniform distribution of other non-canonical residues in the genome

Another type of non-canonical nucleotides of interest for the genome maintenance is ribonucleotides. Despite the significant rNTP vs dNTP discrimination inherent in many DNA polymerases (Williams and Kunkel 2014), the high abundance of rNTP in the cell culminates in frequent incorporation of ribonucleotide during replication with the estimated rate of ~ 1,000,000 insertions per mammalian genome (Reijns et al. 2012). Failure to remove ribonucleotides from DNA, mainly carried out by RNase H2 endonuclease, has been associated with an increase in genome instability. In yeast, RNase H-deficient strain exhibited a greatly elevated rate of spontaneous mutations consisting of 2–5 bp deletions in repeat sequences in a Top1-dependent manner (Kim et al. 2011a; Nick McElhinny et al. 2010). In addition, the accumulation of ribonucleotides in DNA has been correlated with elevation in recombination and gross chromosomal rearrangements in yeast and increased levels of micronuclei, chromosomal rearrangements, interchromosomal translocations, and embryonic lethality in mice (Allen-Soltero et al. 2014; Potenski et al. 2014; Reijns et al. 2012). Most recently, impairment in the ribonucleotide excision repair has been shown to be linked to the increased cytotoxicity of PARP inhibitors (Zimmermann et al. 2018). It would be of particular interest to determine whether a mechanism analogous to the uracil incorporation at highly transcribed genes through unscheduled DNA synthesis could explain the elevated levels of ribonucleotide-dependent mutations observed at highly transcribed genomic regions (Kim et al. 2011a; Nick McElhinny et al. 2010). This is especially intriguing and promising, because, similar to dUTPase, *RNR1*, the gene encoding a subunit of ribonucleotide reductase is tightly regulated in a cell-cycle dependent manner to ensure the maintenance of optimal (rNTP/dNTP) ratio during replication (Elledge and Davis 1990). In considering the role of ribonucleotides in promoting genome instability, their abundance in the genome, and the potential target of ribonucleotide excision repair pathway, it would be useful to understand the factors influencing their density and the distribution patterns in the genome.

## Concluding remarks

The variation in DNA content with non-canonical residues such as uracil and ribonucleotides is a major component of genomic instability. The recent finding that uracil is incorporated into DNA during non-replicative DNA synthesis likely initiated by the transcription-induced endogenous DNA damage leads to the possibility that other non-canonical DNA nucleotides such as ribonucleotides could be incorporated into the DNA by a similar mechanism. This model presents a novel mechanism to account for the variability in the chemical makeup of DNA and suggests that such replication-independent mechanism of incorporation of non-canonical residues could be an important source of mutations in non-proliferating, stationary phase cells or terminally differentiated cells such as the neurons.
